# Notices of the Progress of Experiments on the Influence of Light on the Growth of Plants

**Published:** 1847-02

**Authors:** R. Hunt


*Notices of the Progress of Experiments on the Influence of Light on the Growth of Plants.* By R. Hunt, (Proc. of Brit. Assoc., Sept., 1846, from the Athenæum, Sept. 19.)—The experiments described in the former communications to the Association, had all been confirmed by the results obtained during the past year. It had been found that seeds would not germinate if all the chemical rays were prevented from acting on them—and that the influence of the actinic or chemical rays was such that seeds germinated at a depth below the soil, under the influence of concentrated actinic force, acting on the surface, at which they would not have germinated under the natural conditions. The leaves being developed, the action of the luminous rays then became necessary to effect the decomposition of carbonic acid and the deposition of woody fibre within the plant. Under the joint influence of light and actinism the plant arrived at maturity, and then the calorific, or heat-producing rays were brought more fully into action to produce the ripening of fruit and the development of seed.—*Ibid.*

